# General Intelligence

**Published:** 1844-05

**Authors:** 


### GENERAL INTELLIGENCE.

MEDICAL CLASSES—Session 43, '44.

| | No. of Students. | No. of Graduates. |
|---|---|---|
| University of Pennsylvania | 424 | 148 |
| Jefferson Medical College | 341 | 117 |
| Louisville Medical Institute | 242 | 47 |
| Transylvania University | 214 | 59 |
| Med. College, State of South Carolina | 224 | 82 |
| Medical College of Ohio | 185 | 36 |
| Geneva Medical Institute | 195 | 44 |
| College of Physicians & Surgeons N. Y. | 182 | 32 |
| Albany Medical College | 108 | — |
| Harvard University, Boston | 150 | 17 |
| Kemper College | 90 | 27 |
| Yale College | 60 | 18 |
| Totals | 2415 | 627 |

The number of graduates in the University of the city of New York, was 93; in the Medical College, Richmond, Va., 24: but we have not been able to ascertain the number of students in either of these institutions. From the University of St. Louis, we have heard nothing. In the Rush Medical College of our own city, the number of matriculants was 22,—one only graduating. The organization was effected *but a few weeks* previously to the opening of the Session.—Ed.

---

Notice to Readers and Correspondents.—We are indebted to Prof. Meeker, of LaPorte, Ia., for a communication on the "Epidemic Erysipelas, as it occurred in LaPorte co. Ia.," We regret that we are obliged to postpone its insertion to our next number.

The Medical Examiner has been received from the commencement of the vol. to the latest dates.

The Medical News we have also received from Jan'y. to the last No.

The Cyclopedia of Practical Medicine, Part I. we regret to say, arrived too late to be properly noticed in our present number.

